# Quantfying the disorganization and the core deficit in classical schizophrenia

**DOI:** 10.1192/bjo.2021.754

**Published:** 2021-06-18

**Authors:** Mohan Rathnaiah, Elizabeth B Liddle, Lauren Gascoyne, Jyothika Kumar, Mohammad Zia Ul-Haq Katshu, Catherine Faruqui, Christina Kelly, Malkeet Gill, Sian Robson, Peter Morris, Mathew Brookes, Peter Liddle

**Affiliations:** 1 Institute of Mental Health, University of Nottingham; 2 University of Nottingham; 3 Nottinghamshire Healthcare NHS Foundation Trust

## Abstract

**Aims:**

To derive scores for mental disorganization and impoverishment from commonly used rating scales, and test the hypothesis that disorganization and impoverishment, along with impaired cognition and role-function reflect a latent variable that is a plausible candidate for the putative core deficit.

**Background:**

For more than 100 years, disorganization and impoverishment of mental activity have been recognised as fundamental symptoms of schizophrenia. These symptoms may reflect a core brain process underlying persisting disability. Delusions and hallucinations have been regarded as accessory features. The psychopathological processes predisposing to persisting disability in schizophrenia are poorly understood. The delineation of a core deficit underlying persisting disability would be potentially of great value in predicting outcome and developing improved treatment.

**Method:**

Patients aged 18–55 years were included if: they satisfied DSM IV criteria for schizophrenia or schizoaffective disorder. Healthy controls were recruited by public advertisement and selected to match the patient group in age and sex. Study sample included 39 participants with schizophrenia, 1 with schizoaffective disorder and 44 matched healthy controls. We derived disorganization and impoverishment scores from three symptom scales: PANSS, SSPI and CASH. We computed composite scores for disorganization and for impoverishment and employed Confirmatory Factor Analysis to test the hypothesis that a single factor accounts for the relationships between disorganization, impoverishment, cognitive impairment and impaired role function. We assessed the relationship between this latent “core deficit” and diminished Post Movement Beta Rebound (PMBR), an electrophysiological measure from Magnetoencephalography (MEG), associated with persisting brain disorders.

**Result:**

Fit indices for the single factor model from CFA indicated a good fit: χ2(2) = 1.817, p = .403; RMSEA <.001 GFI = .979. PMBR was significantly reduced in the schizophrenia group compared to healthy controls, t (68) = 3.55, p < .001. Within the patient group, PMBR was significantly and negatively correlated with the CFA factor scores representing the Core Deficit score, r=−.543, p < .01, indicating that high core deficit scores were associated with reduced PMBR. PMBR was significantly correlated with the composite Disorganization score, r=−.521, p < .001.

**Conclusion:**

Our findings demonstrate that the shared variance between impoverishment (psychomotor poverty); disorganization; cognitive impairment; and impaired role function can be accounted for by a latent variable that can reasonably be described as the core deficit of classical schizophrenia. The demonstration that the severity of the putative core deficit is correlated with the reduction in PMBR provides evidence that the core deficit is associated with an identifiable abnormality of brain dysfunction.

